# Poor adherence to guidelines in treatment of fragile and cognitively impaired patients with hip fracture: a descriptive study of 2,804 patients

**DOI:** 10.1080/17453674.2021.1925430

**Published:** 2021-05-12

**Authors:** Christina F Frandsen, Eva N Glassou, Maiken Stilling, Torben B Hansen

**Affiliations:** a University Clinic of Hand, Hip and Knee Surgery, Department of Orthopaedics, Regional Hospital West Jutland; b Department of Quality, Regional Hospital West Jutland; c Department of Clinical Medicine, Aarhus University, Denmark

## Abstract

Background and purpose — Following a hip fracture, most patients will encounter poorer functional outcomes and an increased risk of death. Treatment-monitoring of hip fracture patients is in many countries done by national audits. However, they do not allow for a deeper understanding of treatment limitations. We performed a local evaluation study to investigate adherence to 7 best-practice indicators, and to investigate patient groups at risk of suboptimal treatment.

Patients and methods — 2,804 patients were surgically treated for a hip fracture from 2011 to 2017 at our institution. Data regarding admission, hospital stay, and discharge was prospectively collected, and adherence to the 7 best practice indicators (nerve block, surgical delay, antibiotics, implant choice, thromboprophylaxis, mobilization, and blood transfusions) was analyzed. Patient groups with lower adherence were identified.

Results — 34% of patients received all 7 best practice indicators after considering contraindications; in particular, nerve blocks and thromboprophylaxis displayed low adherence at 61% and 91% respectively. Nursing home residents and patients with cognitive impairment, multiple comorbidities, or low functional levels were at risk of having a lower adherence.

Interpretation — The most dependent patients with cognitive impairment, comorbidities, or low functional levels had lower guideline adherence. This large patient subgroup needs a higher treatment focus and more resources. Our findings are likely similar to those in other national and international institutions.

Hip fractures are a leading cause of disability and mortality among seniors worldwide, with 1-year mortality surpassing 20%. Survivors often experience diminished walking ability, reduced activities of daily living, and loss of independence (Bentler et al. 2009, Dyer et al. 2016). Recent years have seen only minimal improvements in outcomes, such as mortality, which suggest that hip fracture treatment needs improvement (Rogmark 2020). However, patients with hip fracture represent a heterogeneous and fragile patient group with multiple comorbidities, which complicates treatment.

Evidence-based treatment is fundamental to modern medicine, and previous research has demonstrated improved outcomes for patients receiving best practice indicators (Nielsen et al. 2009, Kristensen et al. 2016, Oakley et al. 2017, Farrow et al. 2018). However, most studies are based on process indicators, which give no information on the actual treatment provided; this includes national audits (Sweden’s National Quality Register 2018, Danish Multidisciplinary Hip Fracture Registry 2019, Royal College of Physicians 2019, Australian & New Zealand Hip Fracture Registry 2019). To our knowledge, only a few studies have evaluated direct local adherence to guidelines for patients with hip fracture (Seys et al. 2018, Mcglynn et al. 2003, Sunol et al. 2015). Continuous monitoring through national audits and local studies might detect gaps in the treatment of patients with hip fracture and hopefully secure improvement.

We assessed the degree of adherence to 7 best practice indicators in a local evidence-based guideline for treatment of hip fractures. We expected adherence to increase during the study period as the guideline was incorporated better over time. Furthermore, the study aimed to clarify whether particular patient groups are at risk of significantly lower guideline adherence and hence suboptimal treatment at our institution.

## Patients and methods

### Design

The study is a retrospective analysis of prospectively collected data from a cohort of patients with hip fracture conducted at a department of orthopedic surgery.

### Patients

**Figure 1.**
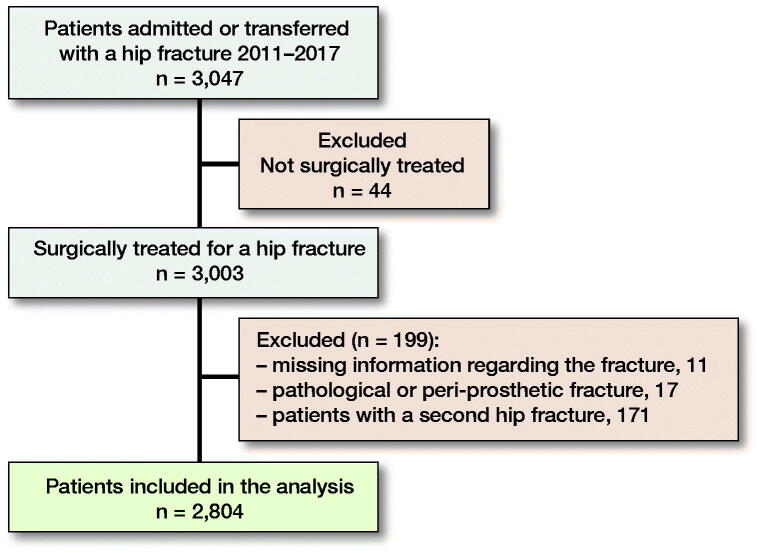
Patient inclusion flowchart.

All patients admitted to our hospital or transferred from other hospitals with a hip fracture between January 2011 and December 2017 were examined for inclusion (n = 3,047). Hip fracture was defined as a femoral neck fracture, an inter-trochanteric fracture, or a sub-trochanteric fracture. Only surgically treated patients were included. Patients with pathological hip fractures or peri-prosthetic fractures were excluded (n = 17). 11 patients with missing data at the start of the study period were also excluded. For patients who suffered a second hip fracture during the study period, only the first hip fracture was included in the analysis (n = 171). 2,804 patients were included in the study (Figure 1).

### Data

All patients were treated according to a well-defined hip fracture guideline at our hospital, which was introduced in January 2011. Simultaneously with the implementation of the guideline, all patients admitted with a hip fracture were prospectively included in our Hip Fracture Database. The database was established in January 2011 to study mortality and morbidity among hip fracture patients at our institution.

During admission, patient characteristics and clinical measures were recorded by a nurse on specified forms for the Hip Fracture Database. Data included weight, height, comorbidity, residency, cognitive impairment, preoperative walking aid, and pre-fracture functional level. At discharge, nurses reported prospectively collected data to the database regarding blood samples, blood transfusions, surgery (date, time, and choice of treatment), pain management (regional block and oral analgesics), and discharge placement.

Comorbidities were assessed by ASA classification. Pre-fracture functional level was estimated using the New Mobility Score (NMS) and was dichotomized into a low pre-fracture functional level (0–5 points) and a high functional level (6–9 points) (Kristensen et al. 2005).

Retrospectively, one researcher (CFF) classified all fractures on the preoperative radiographs (anterior-posterior, lateral view, and pelvic). The radiographs were classified according to the Garden classification for femoral neck fractures and the Evans classification for inter-trochanteric fractures. Posterior tilt was measured on the lateral view for all Garden I–II fractures. No sub-classification for sub-trochanteric fractures was used.

Prior to analysis, we outlined 7 best practice indicators of particular importance in our local hip fracture guideline. National and international recommendations, national audits, and previous literature were reviewed for important indicators (Dansk Ortopaedisk selskab 2008, NICE 2017, Seys et al. 2018, Danish Multidisciplinary Hip Fracture Registry 2019). Indicators were chosen to mirror the different procedural steps and diverse care groups involved in the treatment.

The 7 best practice indicators were as follows:

1. Preoperative block. Defined as the use of either epidural or peripheral nerve block prior to surgery.
2. Surgical delay. Defined as surgery within 24 or 36 hours from admission.
3. Perioperative use of antibiotics.
4. Implant choice. Defined from fracture type and age (Figure 2).

**Figure 2.**
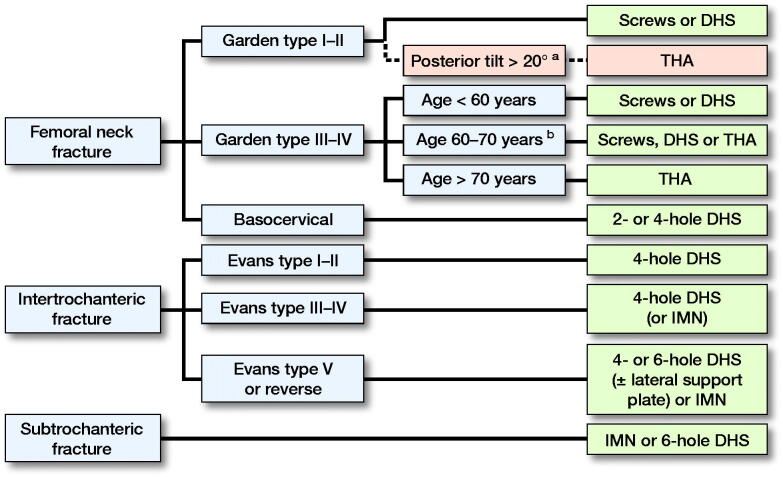
Protocol for implant choice based on fracture type and patient age.
5. Thromboprophylaxis. Defined as injections of low-molecular-weight heparin (LMWH) for at least 7 days with the 1st injection given 6–8 hours after surgery.
6. Postoperative mobilization to standing within 24 hours of surgery.
7. Blood transfusions if postoperative hemoglobin was below 6 mmol/l.

Implant choice was based on recommendations from the Danish Orthopedic Society, and primarily dictated by the fracture type; however, especially for femoral neck fractures the patient’s age was also a determining factor (Dansk Ortopaedisk selskab 2008). Dual mobility total hip arthroplasties (THAs) were used as standard treatment for patients over 70 years with a Garden III and IV fracture. Internal fixation was standard care for younger patients under 60 years due to superior healing potential and to postpone possible revision of a THA in the future. Garden III–IV fractures in patients between 60 and 70 years could be treated with screws, dynamic hip screws (DHSs), or THAs, based on an assessment by the surgeon. Screws or DHSs were used for fractures that could be anatomically reduced and patients without severe comorbidities or severely impaired mobility. For inter-trochanteric fractures, the DHS has been our standard treatment choice; however, for more unstable and complex fractures, intramedullary nails (IMNs) or DHSs with lateral support plate were used, with increasing use of IMNs during the study period. Hemiarthroplasty and external fixation was not performed for hip fractures at our institution.

Data regarding perioperative antibiotics, thromboprophylaxis, and postoperative mobilization was obtained from patient records; data on preoperative pain management, surgical delay, implant choice, and blood transfusions was obtained from our Hip Fracture Database.

Patient records were screened for pre-defined contraindications for each indicator (Table 1).

**Table 1.** Contraindications for each best practice indicator

Factor	Number (%)
1. Preoperative block (n = 1,171)	
Patient declined	155 (13)
No valid contraindications **^a^**	1,016 (87)
2. Surgical delay	
Within 24 hours (n = 738)	
Medical complications **^b^**	128 (17)
Anticoagulation treatment	97 (13)
Death	1 (0.1)
Others	36 (4.9)
No valid contraindication **^a^**	476 (65)
Within 36 hours (n = 376)	
Medical complications **^b^**	99 (26)
Anticoagulation treatment	81 (22)
Death	1 (0.3)
Others	27 (7.2)
No valid contraindication **^a^**	168 (45)
3. Perioperative antibiotics (n = 126)	
Irrelevant antibiotic treatment	33 (26)
No valid contraindication **^a^**	93 (74)
4. Implant choice (n = 349)	
Fracture characteristics	104 (30)
Patient morbidity	61 (18)
Pre-fracture mobility	9 (2.6)
Others	8 (2.3)
No valid contraindication **^a^**	167 (48)
5a. Thromboprophylaxis for 7 days after surgery (n = 211)	
Renal failure	3 (1.4)
Former HIT **^c^**	0 (0.0)
Former bleeding	10 (4.7)
Bridging	88 (42)
Others	39 (20)
No valid contraindication **^a^**	71 (34)
5b. Thromboprophylaxis given 6–8 h after surgery (n = 1,175)	
Given too early	223 (19)
Given too late	691 (59)
Not given the first day postoperatively	136 (12)
Others	22 (1.9)
No valid contraindication **^a^**	103 (8.8)
6. Postoperative mobilization (n = 464)	
No standing abilities prior to surgery	32 (6.9)
Others **^d^**	91 (20)
No valid contraindication **^a,e^**	341 (73)
7. Blood transfusions (n = 90)	
Patient declined	6 (6.7)
Asymptomatic	18 (20)
Others	7 (7.8)
No valid contraindication **^a^**	59 (66)

a Including no reasons given in the patient record or invalid contraindications given.

b For example, cardiac arrhythmias and strokes.

c Heparin-induced thrombocytopenia.

d For example, patient died within 24 h or was transferred to another hospital within 24 h.

e Including patients only mobilized to a sitting position within 24 h.

To investigate whether patient characteristics affected adherence, patients were grouped based on commonly known risk factors for increased mortality and morbidity: age, sex, ASA score, residence, cognitive impairment, fracture type, pre-fracture functional level, and walking aids (Bentler et al. 2009, Smith et al. 2014).

### Statistics

An all-or-none test was performed to clarify the percentages of patients receiving all 7 best practice indicators. Furthermore, adherence was calculated as the proportion of patients who achieved a given number of indicators. A chi-square test was used to assess the hypothesis of no difference in adherence between patient groups to identify groups with statistically significantly lower adherence.

In the statistical analysis, patients with a valid contraindication or missing data were excluded from the adherence analysis for that particular indicator. They remained in the analysis for the other indicators. However, analysis for indicators 3 (perioperative antibiotics) and 5 (thromboprophylaxis) were executed differently. For perioperative antibiotics, the only valid contraindication was if the patient was already in a relevant antibiotic treatment regimen at the time of surgery. These patients were labelled “correctly treated” and remained in the adherence analysis corrected for contraindications. For thromboprophylaxis, patients who were given their 1st injection of LMWH prior to 6 hours or later than 8 hours after surgery were labeled “correctly treated” if they had also received LMWH for 7 days. This was chosen as recent studies have shown that the timing of thromboprophylaxis is not as crucial as had been presumed earlier (Liu et al. 2016, Leer-Salvesen et al. 2018). Only patients with data regarding all 7 best practice indicators were included in the all-or-none test.

Data analyses were performed using STATA 16 computer software (StataCorp, College Station, TX, USA).

### Ethics, funding, and potential conflicts of interest

The study was conducted in accordance with the Declaration of Helsinki and registered by the Danish Data Protection Agency (number 2007-58-0010), which stated no need for written consent according to Danish law. The study has not received any funding. None of the authors has any conflicts of interest to declare.

## Results

2,804 patients were treated for a hip fracture. The mean age was 80 years, and females predominated. The majority lived independently. Almost one-fifth of the patients had cognitive impairment, and half of the population had a low pre-fracture functional level (Table 2).

**Table 2.** Characteristics of the study population at the time of hip fracture (n = 2,804). Values are observed numbers (%) unless ­otherwise stated

Variables	Observed values
Mean age (SD)	80 (11)
Female sex	2,029 (72)
ASA score	
ASA 1	233 (8.3)
ASA 2	1,311 (47)
ASA 3	1,090 (39)
ASA 4	102 (3.6)
ASA 5	1 (0.1)
Missing	67 (2.4)
Pre-fracture residence	
Independent living	2,064 (74)
Institutionalized	736 (26)
Missing	4 (0.1)
Cognitive function	
Cognitively impaired	552 (20)
Not cognitively impaired	2,233 (80)
Missing	19 (0.7)
Fracture type	
Garden type I and II	431 (15)
Garden type III and IV	977 (35)
Stable intertrochanteric	572 (20)
Unstable intertrochanteric	680 (24)
Subtrochanteric	57 (2.0)
Basocervical	81 (2.9)
Missing	6 (0.2)
Pre-fracture mobility **^a^**	
Low NMS	1,405 (50)
High NMS	1,274 (45)
Missing	125 (4.5)
Walking aids	
None	1,047 (37)
Assisted walking	1,484 (53)
No walking ability	79 (2.8)
Missing	194 (6.9)

a Pre-fracture mobility was assessed by New Mobility Score (NMS) with 0–5 points labelled as low and 6–9 points as high.

### Total study period

17% of patients received all 7 best practice indicators. The lowest adherence was found for preoperative block and thromboprophylaxis. The indicators with the highest degree of adherence were perioperative antibiotics and implant choice (Table 3). Overall adherence increased to 34% after considering contraindications, primarily due to increased adherence to thromboprophylaxis (Table 3). Furthermore, 65% of patients in fact fulfilled 6 or more indicators (Table 4).

**Table 3.** Observed adherence to the guideline for each of the 7 best practice indicators and all-or-none adherence to all 7 best practice indicators, listed as total number and observed numbers (%)

Factor	Adherence to guideline	Corrected for contraindications
1. Preoperative block	2,793	2,638
	1,607 (57)	1,607 (61)
2. Surgical delay		
within 24 hours	2,786	2,524
	2,048 (74)	2,048 (81)
within 36 hours	2,787	2,579
	2,411 (87)	2,411 (93)
3. Perioperative antibiotics	2,759	2,759
	2,633 (95)	2,666 (97)
4. Implant choice	2,804	2,613
	2,446 (87)	2,446 (94)
5. Thromboprophylaxis	2,787	2,640
	1,538 (55)	2,394 (91)
6. Postoperative mobilization	2,675	2,552
	2,211 (83)	2,211 (87)
7. Blood transfusions	718	687
	628 (87)	628 (91)
All-or-none	2,629	2,028
	442 (17)	684 (34)

**Table 4.** Percentage of patients fulfilling 3, 4, 5, 6, or 7 best practice indicators (n = 1,946)

Number of indicators fulfilled	Observed numbers (%)
3	7 (0.4)
4	53 (2.6)
5	319 (17)
6	883 (45)
7	684 (35)

### Annual adherence

**Figure 3.**
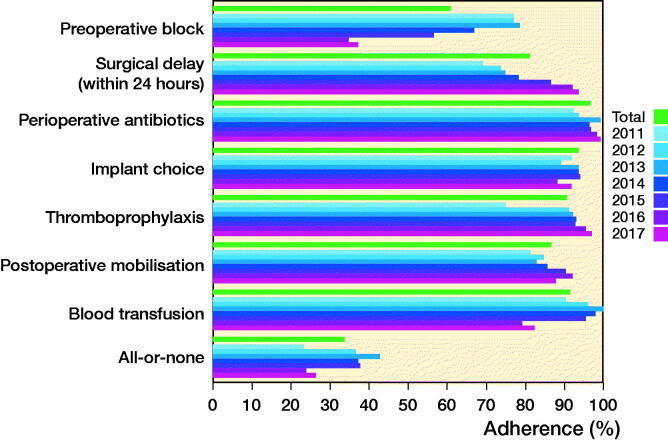
Adherence to the individual best practice indicators and overall adherence in percentages divided into the different years of the study period after taking contraindications into consideration. ^a^Posterior tilt > 20° in the lateral view of Garden I–II fractures resulted in the recommended treatment changing to a THA. However, as this was part of the adjustment after contraindications it is shown in dotted lines but included in the figure for clarification. ^b^Individual assessment of each patient’s comorbidity, pre-fracture mobility, and radiograph to determine best treatment option, favoring screws or DHS in fractures that can be anatomically reduced, and patients without severe comorbidities and severely impaired pre-fracture mobility. DHS = dynamic hip screw. IMN = intramedullary nail. THA = total hip arthroplasty.

Adherence to individual best practice indicators and overall adherence for each year in the study period are displayed in Figure 3 (the data is shown after considering contraindications). Preoperative block showed a decline in adherence during the 7-year period, and blood transfusions dropped in 2016 and 2017. Both declines had an impact on overall adherence, which decreased in 2016 and 2017 to 24% and 26%, respectively. Data is also shown in Table 5 (see Supplementary data).

**Table 5.** Adherence in the individual years 2011–2017 (only adherence corrected for contraindications is displayed). Values are observed numbers and % of patients fulfilling the given indicator

	2011 n = 420	2012 n = 408	2013 n = 428	2014 n = 364	2015 n = 377	2016 n = 403	2017 n = 404
1. Preoperative block	292 (77)	294 (77)	308 (79)	224 (70)	204 (57)	137 (35)	146 (37)
2. Surgical delay (within 24 hours)	253 (69)	269 (74)	288 (75)	251 (78)	295 (87)	340 (92)	351 (94)
3. Perioperative antibiotics	359 (92)	378 (94)	419 (99)	350 (96)	362 (97)	395 (98)	401 (99)
4. Implant choice	342 (92)	331 (89)	367 (92)	319 (94)	310 (94)	345 (88)	346 (92)
5. Thromboprophylaxis	303 (75)	362 (91)	378 (92)	317 (93)	328 (93)	359 (96)	345 (97)
6. Postoperative mobilization	231 (81)	322 (85)	334 (83)	291 (86)	319 (90)	355 (92)	336 (88)
7. Blood transfusions	85 (90)	97 (96)	102 (100)	93 (98)	84 (96)	83 (79)	84 (82)
All-or-none	60 (23)	106 (37)	135 (43)	94 (37)	106 (38)	75 (24)	76 (26)

### Adherence in subgroups

No difference in overall adherence was found when comparing adherence in patient groups in relation to age groups, sex, or fracture type. However, nursing home residents, cognitively impaired patients, patients with a low pre-fracture functional level, and patients with high comorbidity (ASA > 4) were at risk of receiving insufficient treatment (Table 6, see Supplementary data).

**Table 6.** Chi-square test for no difference in adherence based on characteristics of the study population. Values are observed numbers (%)

		All or no adherence		
Characteristics	Total	Fulfilled	Not fulfilled	p-value
Age (n = 2,028)				0.6
≤ 55 years	127 (6.3)	45 (35)	82 (65)	
56–69 years	256 (13)	92 (36)	164 (64)	
≥ 70 years	1,645 (81)	547 (33)	1,098 (67)	
Sex (n = 2,028)				0.9
Female	1,364 (67)	461 (34)	903 (66)	
Male	664 (33)	223 (34)	441 (66)	
ASA (n = 1,991)				0.008
ASA 1	192 (9.7)	67 (35)	125 (65)	
ASA 2	1,018 (51)	373 (37)	645 (63)	
ASA 3	731 (37)	222 (30)	509 (70)	
ASA 4	50 (2.5)	10 (20)	40 (80)	
Residence (n = 2,027)				0.004
Independently	1,537 (76)	545 (35)	992 (64)	
Institution	490 (24)	139 (28)	351 (72)	
Cognitive impairment (n = 2,017)				0.01
No cognitive impairment	1,647 (82)	578 (35)	1,069 (65)	
Cognitive impairment	370 (18)	104 (28)	266 (73)	
Fracture type (n = 2,026)				0.1
Garden I + II	313 (15)	90 (29)	223 (71)	
Garden III + IV	703 (35)	256 (36)	447 (64)	
Stable intertrochanteric	419 (21)	149 (36)	270 (64)	
Unstable intertrochanteric	499 (25)	161 (32)	338 (68)	
Subtrochanteric	40 (2.0)	9 (23)	31 (77)	
Basocervical	52 (2.6)	19 (37)	33 (63)	
Pre-fracture functional level **^a^** (n = 1,951)			< 0.001	<0.001
Low NMS	968 (50)	284 (29)	684 (71)	
High NMS	983 (50)	383 (39)	600 (61)	
Walking aids (n = 1,882):				0.01
Independently	784 (42)	292 (37)	492 (63)	
Assisted walking	1,062 (56)	326 (31)	736 (69)	
No walking ability	36 (1.9)	12 (33)	24 (67)	

a NMS, see Table 2

## Discussion

Our most important finding was that only one-third of hip fracture patients at our institution fulfilled all 7 best practice indicators; however, the majority of patients received 6 or more indicators. The study also suggests lower adherence among patients with multiple comorbidities, cognitive impairment, low pre-fracture functional level, and nursing home residency. Surprisingly, adherence did not increase during the study period because of declining adherence to the indicators “preoperative pain management” and “blood transfusion” in 2016 and 2017. The decrease in adherence to preoperative pain management might be explained by a major organizational change in 2016, whereby admissions past 10 pm were performed by medical doctors rather than orthopedic surgeons or ER doctors. Medical doctors have less experience in administering regional blocks at our institution, which may cause fewer blocks to be administered. For blood transfusions, decreasing adherence over the study period might be due to the 2015 introduction of a new guideline setting lower hemoglobin limits (< 5.6 mmol/L) for transfusion (Danish Healthcare System 2015). Although the transfusion guideline was not specific for patients with hip fracture, it likely influenced the use of blood transfusions for this patient group as well.

Other local evaluation studies have similarly found suboptimal care for patients with hip fracture, some with an overall adherence of 0% (Mcglynn et al. 2003, Sunol et al. 2015, Farrow et al. 2018, Seys et al. 2018). Contrary to local evaluations, most national audits show a high level of adherence (Sweden’s National Quality Register 2018, Danish Multidisciplinary Hip Fracture Registry 2019, Australian & New Zealand Hip Fracture Registry 2019). Local evaluation studies should be seen as a supplement to national audits as they can provide a deeper understanding of treatment gaps. Surgical delay may serve as a good illustrator. National audits can only give the results, whereas local studies can point to capacity issues or patient comorbidities as the reason for low adherence. This will identify what steps are needed to increase adherence. The same is true for implant choice as national audits can only give the proportion of patients receiving the different implants; they cannot determine whether the choice of implant was the right one.

In previous years, our institution has demonstrated high adherence to the national Danish audit, and similar adherence was found in the present study for matching indicators (Danish Multidisciplinary Hip Fracture Registry 2019). However, when investigating overall adherence, only one-third of patients obtained full treatment. This indicates that care for patients with hip fracture may also be suboptimal at other hospitals despite high adherence to the national audit, underlining the need for local evaluation. Nevertheless, national audits play an important role in monitoring treatment as adherence to national indicators has shown reduced mortality and readmission (Nielsen et al. 2009, Kristensen et al. 2016). Supplementing national audits with local studies in the future may inform future initiatives to improve hip fracture treatment.

For the individual best practice indicators, the most surprising results were low adherence for preoperative pain management and thromboprophylaxis. Preoperative pain management will be a future focus area because optimal pain management, especially the use of preoperative blocks, may improve recovery by reducing the use of opioids and by reducing nausea and dizziness, while helping in improving mobilization and nutrition (Guay et al. 2017). Thromboprophylaxis had low adherence before considering contraindications. Low adherence was especially due to bridging, where patients did not receive LMH for 7 days, and patients receiving the 1st injection before 6 hours or later than 8 hours after surgery. However, after the start of the study, anticoagulation therapy has changed. Previous studies have shown that the timing of the thromboprophylaxis is of less importance (Liu et al. 2016, Leer-Salvesen et al. 2018), and with the emergence of new anticoagulation strategies, bridging has become more frequent. Consequently, a revision of the guideline is required. The change in anticoagulation therapy also had an impact on the best practice indicator “surgical delay.” Especially in the early stages of the study, vitamin K antagonists (VKA) and novel oral anticoagulants (NOAC) posed a challenge. Surgery was delayed when patients did not respond to vitamin K within 24 hours or due to the initial recommendation of a 24-hour pause from NOAC. However, more recent studies have demonstrated that operating regardless of anticoagulation therapy is, indeed, safe (Schuetze et al. 2019). Despite these delays, our study shows an impressive level of adherence compared with international standards, where most guidelines have a 36-or 48-hour deadline and most national audits show lower fulfilment than in this study (Sweden’s National Quality Register 2018, Australian & New Zealand Hip Fracture Registry 2019, Royal College of Physicians 2019). The increased adherence is probably due to an organizational change with more experienced surgeons and operating rooms functioning outside standard working hours.

Implant choice adherence was in line with that reported in other studies (Palm et al. 2012). Most patients had valid contraindications when the guideline was not followed. Contraindications for implant choice were fracture characteristics, primarily a posterior tilt above 20 degrees on the lateral radiograph for Garden I and II fractures (Table 1). Here we opted for a THA instead of screws for patients to reduce the risk of reoperation (Palm et al. 2009). Patient morbidity and mobility describe situations such as young patients with severe osteoporosis or mental handicaps, or patients with no standing or walking ability. In such cases, guideline adherence would be deselected to reduce the risk of reoperation or having to perform extensive surgery. Other contraindications were patients declining the recommended implant.

Our study has several strengths. A major strength is a high level of external validity owing to inclusion of all consecutive patients with a hip fracture admitted to the department, including patients with severe cognitive impairment and multiple comorbidities, reducing selection bias. Another strength is the use of prospectively collected or documented data, reducing the risk of recall bias.

As with most studies, the design of our study is subject to limitations. First, we have missing data in relation to some variables. If information concerning antibiotics, thromboprophylaxis, postoperative mobilization, and the predefined contraindications were not documented during admission and therefore not available in patient records, these variables were interpreted as missing. This interpretation would have led to an underestimation of the adherence to the guideline. Despite this approach, we had a high degree of data completeness. Second, our study was limited by being a single-center study. While this ensured that patients were treated similarly, it also meant together with the descriptive nature of the study that we can only be sure the results are valid for our institution. However, these results could be true for other institutions, as previous studies have found similar results and the national audit had comparable adherence for matching indicators (Mcglynn et al. 2003, Sunol et al. 2015, Farrow et al. 2018, Seys et al. 2018, Danish Multidisciplinary Hip Fracture Registry 2019). Third, a lack of consensus on which best practice indicators to use as predictors for adherence in hip fracture treatment hampers comparison with other results. Previous studies have used a wide variety of indicators from procedures (orthopedic or geriatric assessment of patients), timing (of surgery, postoperative mobilization, admission to orthopedic wards, or assessment by senior doctors) and medical indicators (antibiotics, thromboprophylaxis, and pain management). A Delphi study was conducted by Seys et al. (2018), to identify indicators of importance in the patients with hip fracture. 4 of the 7 best practice indicators in our study were found to be important for treatment of patients with hip fracture in the Delphi study (surgical delay, antibiotics, thromboprophylaxis, and postoperative mobilization) and 1 was found to be of less importance (preoperative pain management). Further research should be conducted to establish general consensus on which best practice indicators to use, which will ease comparison between studies. Risk assessment for pressure ulcers and malnutrition may be important indicators in improving treatment; furthermore, indicators regarding the period after discharge, such as osteoporosis treatment, fall prophylaxis, and rehabilitation, should be considered in future studies.

### Conclusion

In summary, we found that despite high adherence to individual best practice indicators, overall adherence is surprisingly low at our institution, especially among fragile and cognitively impaired patients. A local evaluation study, such as ours, can be used in the clinic to identify patient groups or treatment steps that need improvement and to deepen our understanding of treatment gaps.

## Supplementary data

Tables 5 and 6 are available as supplementary data in the online version of this article, http://dx.doi.org/10.1080/17453674.2021.1925430

## References

[CIT0001] Australian & New Zealand Hip Fracture Registry. Annual Report 2019; 2019. https://anzhfr.org/2019-annual-report/

[CIT0002] Bentler S E, Liu L, Obrizan M, Cook E A, Wright K B, Geweke J F, Chrischilles E A, Pavlik C E, Wallace R B, Ohsfeldt R L, Jones M P, Rosenthal G E, Wolinsky F D. The aftermath of hip fracture: discharge placement, functional status change, and mortality. Am J Epidemiol 2009; 170 (10): 1290–9. 19808632 10.1093/aje/kwp266 PMC2781759

[CIT0003] Danish Healthcare System. Vejledning om blodtransfusion. doi: j.rn: 572109/2; 2015.

[CIT0004] Danish Multidisciplinary Hip Fracture Registry. Danish Multidisciplinary Hip Fracture Registry, Nationalrapport; 2019. https://www.sundhed.dk/content/cms/62/4662_hofterapport.pdf.

[CIT0005] Dansk Ortopaedisk selskab. Reference program for Patienter Med Hoftebrud; 2008. https://www.ortopaedi.dk/fileadmin/Guidelines/Referenceprogrammer/Referenceprogram_for_patienter_med_hoftebrud2008.pdf.

[CIT0006] Dyer S M, Crotty M, Fairhall N, Magaziner J, Beaupre L A, Cameron I D, Sherrington C. A critical review of the long-term disability outcomes following hip fracture. BMC Geriatrics 2016; 16: 158. 27590604 10.1186/s12877-016-0332-0 PMC5010762

[CIT0007] Farrow L, Hall A, Wood A D, Smith R, James K, Holt G, Hutchison J, Myint P K. Quality of care in hip fracture patients: the relationship between adherence to national standards and improved outcomes. J Bone Joint Surg Am 2018; 100: 751–57. 29715223 10.2106/JBJS.17.00884

[CIT0008] Guay J, Parker M J, Griffiths R, Kopp S. Peripheral nerve blocks for hip fractures (review). Cochrane Database Syst Rev 2017; 5(5): CD001159. 28494088 10.1002/14651858.CD001159.pub2 PMC6481480

[CIT0009] Kristensen M T, Foss N B, Kehlet H. Timed Up & Go og New Mobility Score til praediktion af funktion seks måneder efter hoftefraktur [Timed Up and Go and New Mobility Score as predictors of function six months after hip fracture]. Ugeskr Laeger 2005; 167(35): 3297–3300. PMID: 16138973. 16138973

[CIT0010] Kristensen P K, Thillemann T M, Søballe K, Johnsen S P. Are process performance measures associated with clinical outcomes among patients with hip fractures? A population-based cohort study. Int J Qual Health Care 2016; 28(6): 698–708. 27591269 10.1093/intqhc/mzw093

[CIT0011] Leer-Salvesen S, Dybvik E, Engesaeter L B, Dahl O E, Gjertsen J. Low-molecular-weight heparin for hip fracture patients treated with osteosynthesis: should thromboprophylaxis start before or after surgery? An observational study of 45,913 hip fractures reported to the Norwegian Hip Fracture Register. Acta Orthop 2018; 89(6): 615–21. 30328746 10.1080/17453674.2018.1519101 PMC6300732

[CIT0012] Liu Z, Han N, Xu H, Fu Z, Zhang D, Wang T, Jiang B. Incidence of venous thromboembolism and hemorrhage related safety studies of preoperative anticoagulation therapy in hip fracture patients undergoing surgical treatment: a case-control study. BMC Musculoskeletal Disorders 2016; 17(76): 1–8. 26873584 10.1186/s12891-016-0917-y PMC4752756

[CIT0013] Mcglynn E A, Asch S M, Adams J, Keesey J, Hicks J, Decristofaro A, Kerr E A. The quality of health care delivered to adults in the United States. N Engl J Med 2003; 348: 2635–45. 12826639 10.1056/NEJMsa022615

[CIT0014] NICE. National Institute for Health and Clinical Excellence (NICE) (2012) Management of hip fractures in adults; 2017. https://www.nice.org.uk/guidance/qs16.

[CIT0015] Nielsen K A, Jensen N C, Jensen C M, Thomsen M, Pedersen L, Johnsen S P, Ingeman A, Bartels P D, Thomsen R W. Quality of care and 30-day mortality among patients with hip fractures: a nationwide cohort study. BMC Health Serv Res 2009; 9: 186. 19822018 10.1186/1472-6963-9-186 PMC2768699

[CIT0016] Oakley B, Nightingale J, Moran C G, Moppett I K. Does achieving the best practice tariff improve outcomes in hip fracture patients? An observational cohort study. BMJ Open 2017; 7(2): e014190. 10.1136/bmjopen-2016-014190 PMC5293976 28167748

[CIT0017] Palm H, Gosvig K, Krasheninnikoff M, Jacobsen S, Gebuhr P. A new measurement for posterior tilt predicts reoperation in undisplaced femoral neck fractures 113 consecutive patients treated by internal fixation and followed for 1 year. Acta Orthop 2009; 80(3): 303–7. 19634021 10.3109/17453670902967281 PMC2823202

[CIT0018] Palm H, Krasheninnikoff M, Holck K, Lemser T, Foss N B, Jacobsen S, Kehlet H, Gebuhr P. A new algorithm for hip fracture surgery: reoperation rate reduced from 18% to 12% in 2,000 consecutive patients followed for 1 year. Acta Orthop 2012; 83(1): 26–30. 22248165 10.3109/17453674.2011.652887 PMC3278653

[CIT0019] Rogmark C. Further refinement of surgery will not necessarily improve outcome after hip fracture. Acta Orthop 2020; 91(2): 123–24. 31913741 10.1080/17453674.2019.1706936 PMC7144235

[CIT0020] Royal College of Physicians. The National Hip Fracture Database; 2019. https://www.nhfd.co.uk/.

[CIT0021] Schuetze K, A Eickhoff, Dehner C, Gebhard F, Richter P H. Impact of oral anticoagulation on proximal femur fractures treated within 24 h: a retrospective chart review. Injury 2019; 50(11): 2040–4. 31543315 10.1016/j.injury.2019.09.011

[CIT0022] Seys D, Sermon A, Sermeus W, Panella M, Bruyneel L, Boto P. Recommended care received by geriatric hip fracture patients: where are we now and where are we heading? Arch Orthop Trauma Surg 2018; 138: 1077–87. 29704045 10.1007/s00402-018-2939-4

[CIT0023] Smith T, Pelpola K, Ball M, Ong Al, Myint P K. Pre-operative indicators for mortality following hip fracture surgery: a systematic review and meta-analysis. Age Ageing 2014; 43(4): 464–71. 24895018 10.1093/ageing/afu065

[CIT0024] Sunol R, Wagner C, Arah O A, Kristensen S. Implementation of departmental quality strategies is positively associated with clinical practice: results of a multicenter study in 73 hospitals in 7 European countries. PLoS One 2015; 10(11):: e0141157. 26588842 10.1371/journal.pone.0141157 PMC4654525

[CIT0025] Sweden’s National Quality Register. Annual Report 2018; 2018. https://rikshoft.se/wpcontent/uploads/2019/11/rikshoft_rapport2018_191023.pdf.

